# Supplementary figures and images for: Bicc1 ribonucleoprotein complexes specifying organ laterality are licensed by ANKS6-induced structural remodeling of associated ANKS3
Source: PLoS Biol. 2023 Sep 21;21(9):e3002302. doi: 10.1371/journal.pbio.3002302 (PMC10513324; doi:10.1371/journal.pbio.3002302)

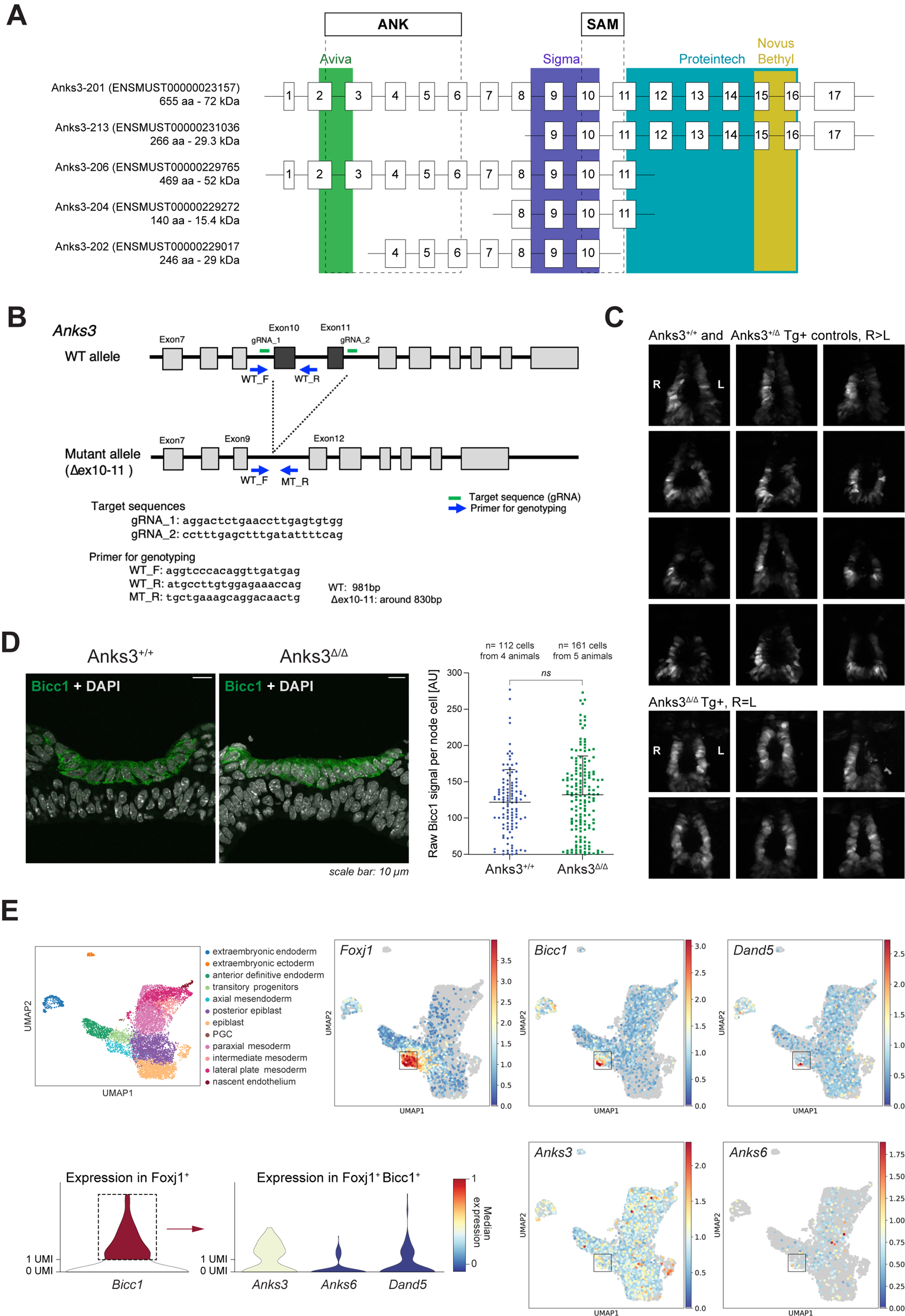

Supplement: S1 Fig — (A) Alternative splicing of mouse Anks3 annotated in Ensembl [38] and coding regions of domains used to raise the commercial antibodies indicated. (B) Anks3 mutant mice were generated using 2 gRNAs (green bars) to delete exons 10 and 11. Positions of PCR primers (blue arrows) used for genotyping are indicated. (C) Fluorescence of dsVenus at the node of Anks3Δ/Δ embryos and control litter mates harboring the NDE-Hsp-dsVenus-Dand5 3′ UTR transgene (TG+) at 3- to 5-somite stages. Pictures correspond to individual values presented in the graphs of Fig 1C prior to correction for variation of laser power and voltage settings. (D) Immunofluorescence staining of Bicc1 (green) in node cells of Anks3+/+ and Anks3Δ/Δ embryo at E8.0. Quantification of Bicc1 immunofluorescence intensities is shown in the graph on the right as the means + SD from 112 and 161 cells from 4 Anks3+/+ and 5 Anks3Δ/Δ animals, respectively. ns: nonsignificant (Student’s t test). (E) UMAP plots showing the expression of the node cell markers Foxj1 and Bicc1 (n = 1,363 cells), corresponding to the axial mesendoderm population in a public scRNA-seq dataset [24]. Cell containing at least 1 unique molecular identifier corresponding to Bicc1 were considered to be Bicc1+. Dand5, Anks3, and Anks6 mRNA expression is also observed in the axial mesendoderm population (shown in UMAPs by the square box and represented by violin plots). Underlying data can be found in the S1 Raw Values file. (TIF) [file pbio.3002302.s001.tif]

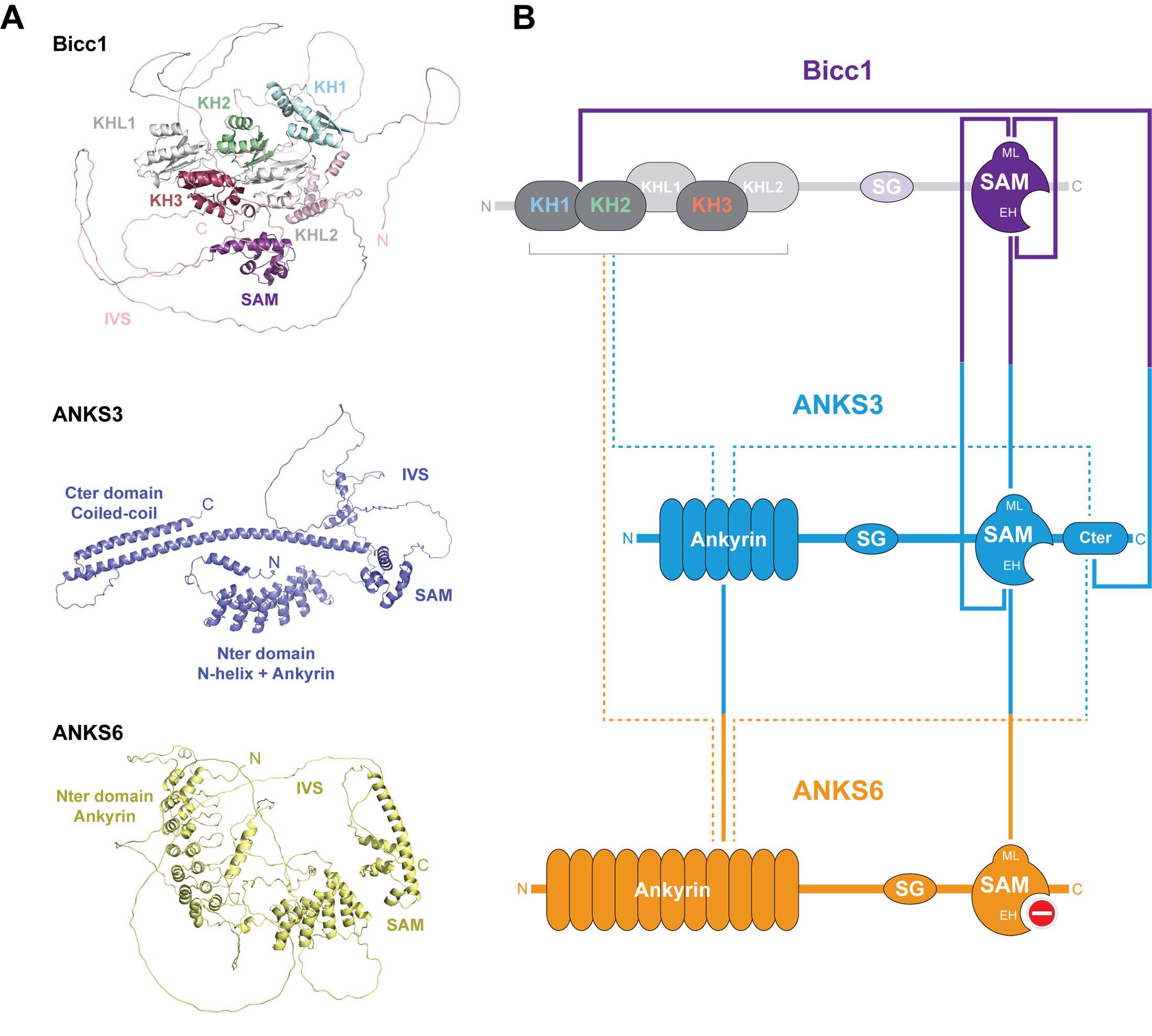

Supplement: S2 Fig — (A) Model structures of the indicated proteins released by AlphaFold. Structured domains are annotated. Serine/glycine (SG)-rich linker regions, including the intervening sequence (IVS) of Bicc1 are predicted to be intrinsically disordered. (B) Cartoon depicting the multivalent interactions in the Bicc1-ANKS3-ANKS6 protein network. Connecting straight lines indicate validated protein–protein interactions. The one-way sign indicates that the EH surface of the ANKS6 does not bind the SAM domains of either Bicc1 or ANKS3. (TIF) [file pbio.3002302.s002.tif]

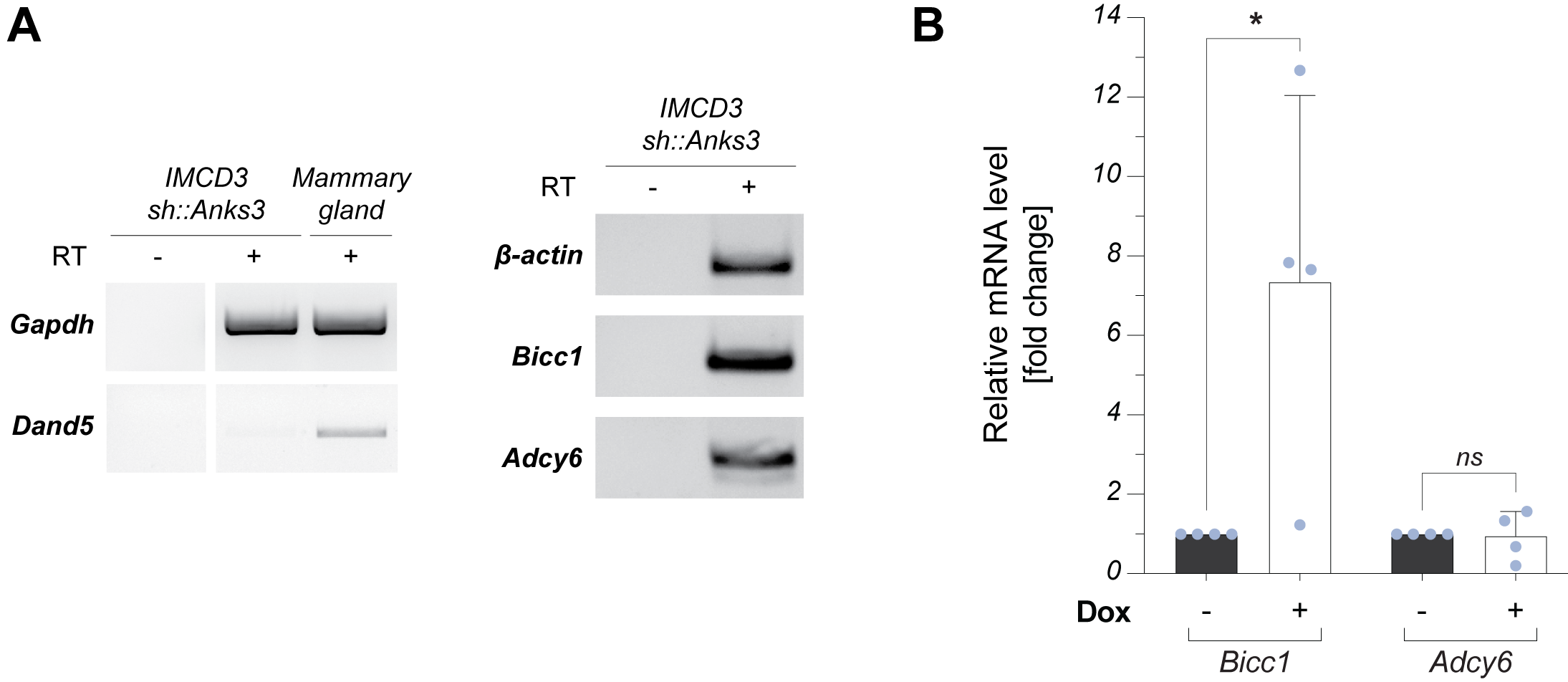

Supplement: S3 Fig — (A) RT-PCR detection of endogenous mRNAs in the input fraction of untreated IMCD3-sh::ANKS3 cells used for co-immunoprecipitation in Fig 5. cDNA from mouse mammary gland was used as a positive control for the expression of Dand5 based on a search for DAND5-expressing cell lines and tissues in the Human Protein Atlas database (https://www.proteinatlas.org/ENSG00000179284-DAND5/tissue). (B) Expression levels of Bicc1 and Adcy6 mRNAs relative to β-actin measured by RT-qPCR analysis in IMCD3 before and after doxycycline-induced Anks3 depletion. The values are expressed as the fold change normalized to the untreated condition. These data are related to the co-immunoprecipitation results shown in Fig 5. Data are means + SD from 4 independent experiments. ns: nonsignificant, *p < 0.05, **p < 0.01, ***p < 0.001 (Student’s t test). Underlying data can be found in the S1 Raw Images and S1 Raw Values files. (TIF) [file pbio.3002302.s003.tif]

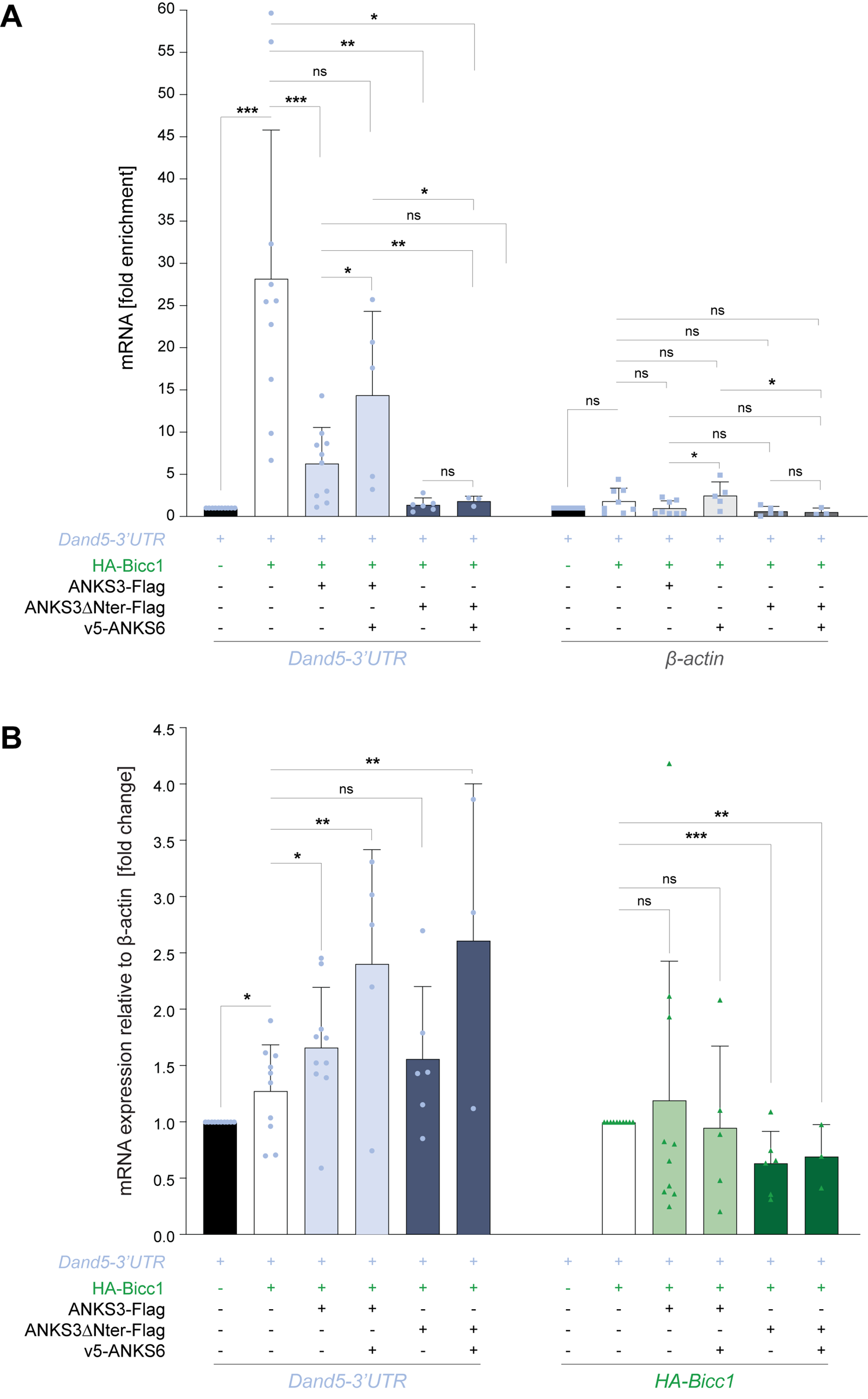

Supplement: S4 Fig — (A) Additional analysis of the RT-qPCR data shown in Fig 6B. Ratios of co-immunoprecipitated mRNAs over the input, normalized to the amount of HA-Bicc1 in the IP fraction and then expressed relative to the corresponding control condition without HA-Bicc1. β-actin mRNA served as a negative control to visualize the level of unspecific RNA binding by Bicc1. (B) Expression level of the dsVenus-Dand5 3′ UTR and HA-Bicc1 mRNAs relative to β-actin measured by RT-qPCR analysis in transfected HEK293T cells. These data are related to the co-immunoprecipitation results shown in Fig 6B. Data are means + SD from between 3 to 10 independent experiments. ns: nonsignificant, *p < 0.05, **p < 0.01, ***p < 0.001 (Student’s t test). Underlying data can be found in the S1 Raw Values file. (TIF) [file pbio.3002302.s004.tif]
