# Supplementary material for: Bicc1 ribonucleoprotein complexes specifying organ laterality are licensed by ANKS6-induced structural remodeling of associated ANKS3
Source: PLoS Biol. 2023 Sep 21;21(9):e3002302. doi: 10.1371/journal.pbio.3002302 (PMC10513324; doi:10.1371/journal.pbio.3002302)
Supplement: S2 Table — (PDF) [file pbio.3002302.s006.pdf]

Species	Primer Name	Sequence
Human	BR140-hBact-F	ACAGAGCCTCGCCTTTGCC
Human	BR141-hBact-R	CTCCATGCCCAGGAAGGAAGG
Mouse	BR144-mAC6-F	ACCGAATACAGGTGACTACGGAC
Mouse	BR145-mAC6-R	GTCCCCTCCATCGCCATTCT
Mouse	BR150-mBact-F	AGATCAAGATCATTGCTCCTCCTGA
Mouse	BR151-mBact-R	TCGCCTTCACCGTTCCAGTT
Mouse	BR347-mDand5-F	GCTGAGCATCCTAGAGGAATGC
Mouse	BR402-mDand5-R	TAAACCCATGACTGGGGGACCATGTCTAG
Mouse	BR350-mAnks3-F	TGTTAGCCAGGCAGTTTGGT
Mouse	BR351-mAnks3-R	GAGGGACTTGCTCATAGGTGG
Mouse	BR364-mBicc1A-F	CAACTGGAGAGACCGGAATGGA
Mouse	BR365-mBicc1A-R	CTTAGCTCTGAGATTGCCAGCAGC
