# Supplementary material for: Bicc1 ribonucleoprotein complexes specifying organ laterality are licensed by ANKS6-induced structural remodeling of associated ANKS3
Source: PLoS Biol. 2023 Sep 21;21(9):e3002302. doi: 10.1371/journal.pbio.3002302 (PMC10513324; doi:10.1371/journal.pbio.3002302)
Supplement: S1 Raw Images — (PDF) [file pbio.3002302.s007.pdf]

Figure 2 - source data

Figure 2B

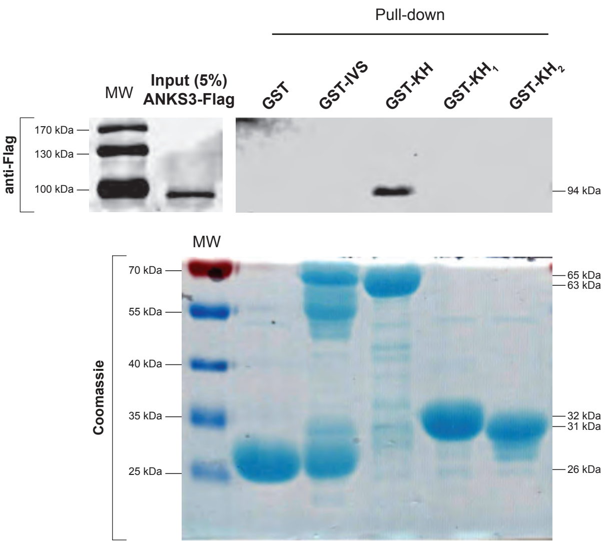

Figure 2F

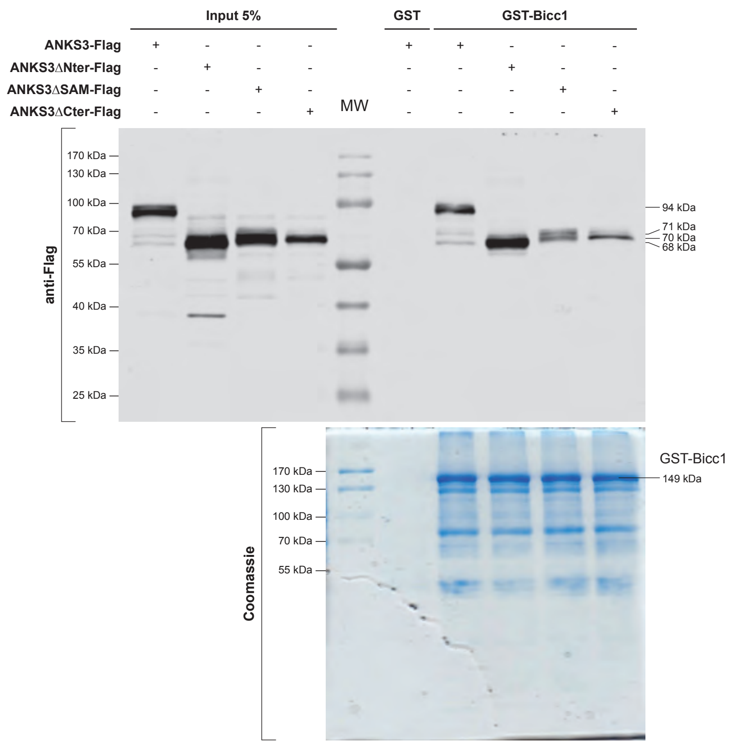

Figure 2G

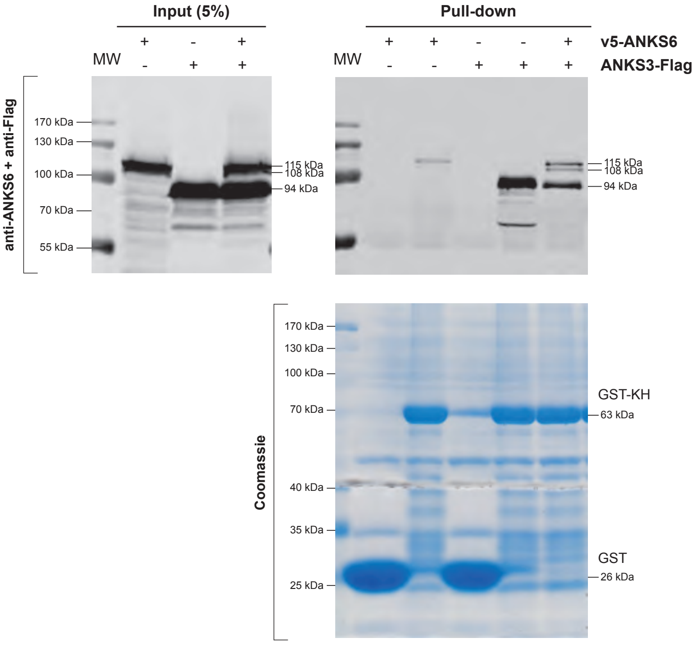

Figure 3 - source data

Figure 3B

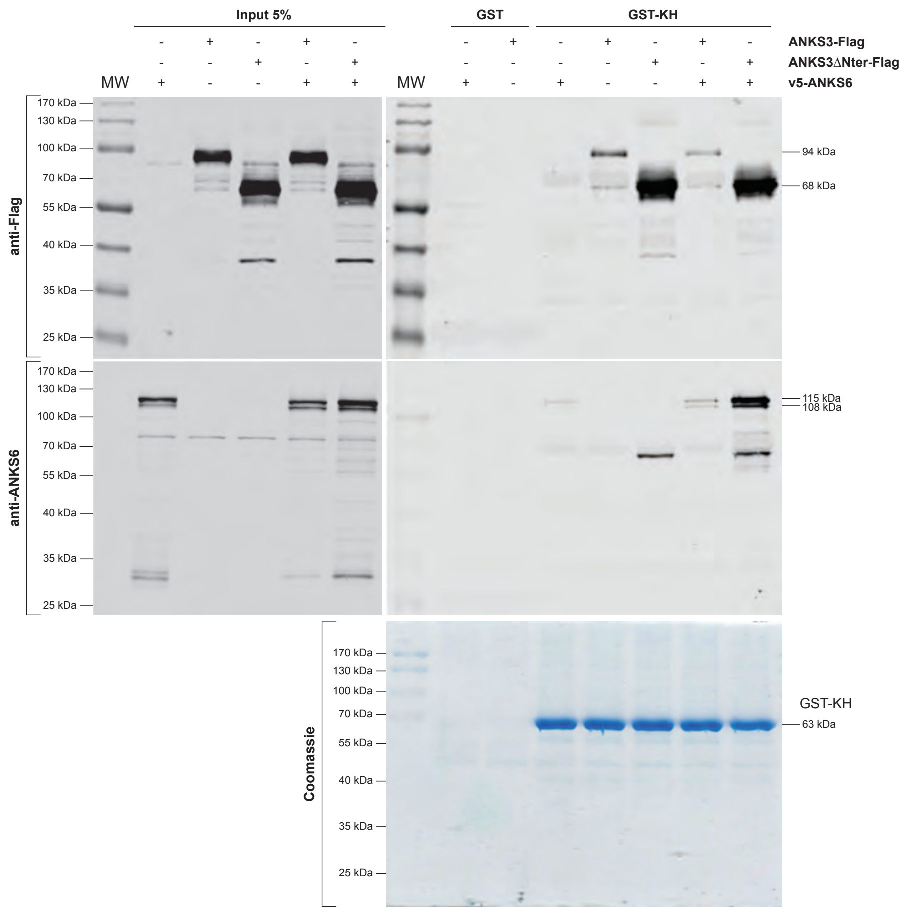

Figure 4 - source data

Figure 4A

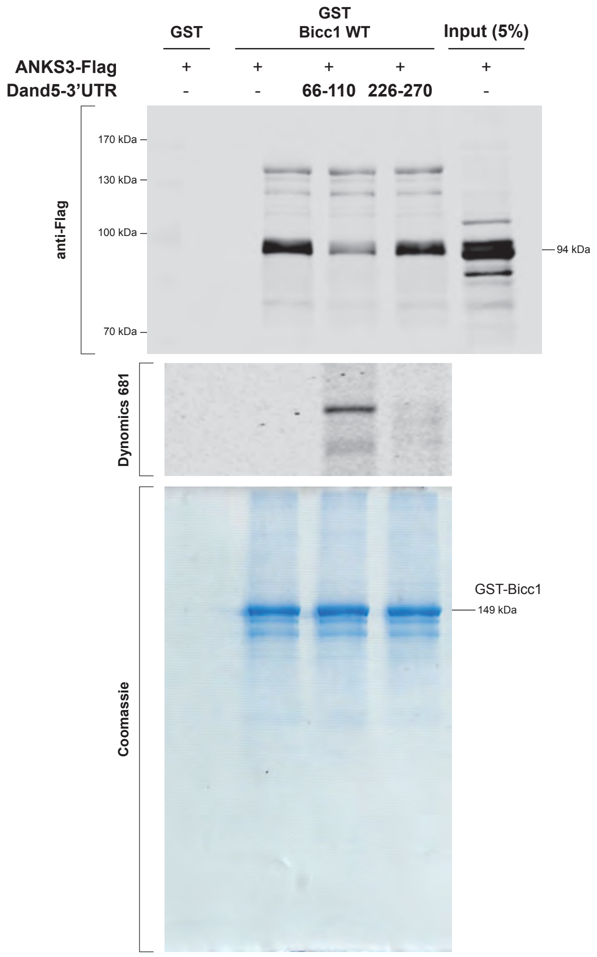

Figure 4C

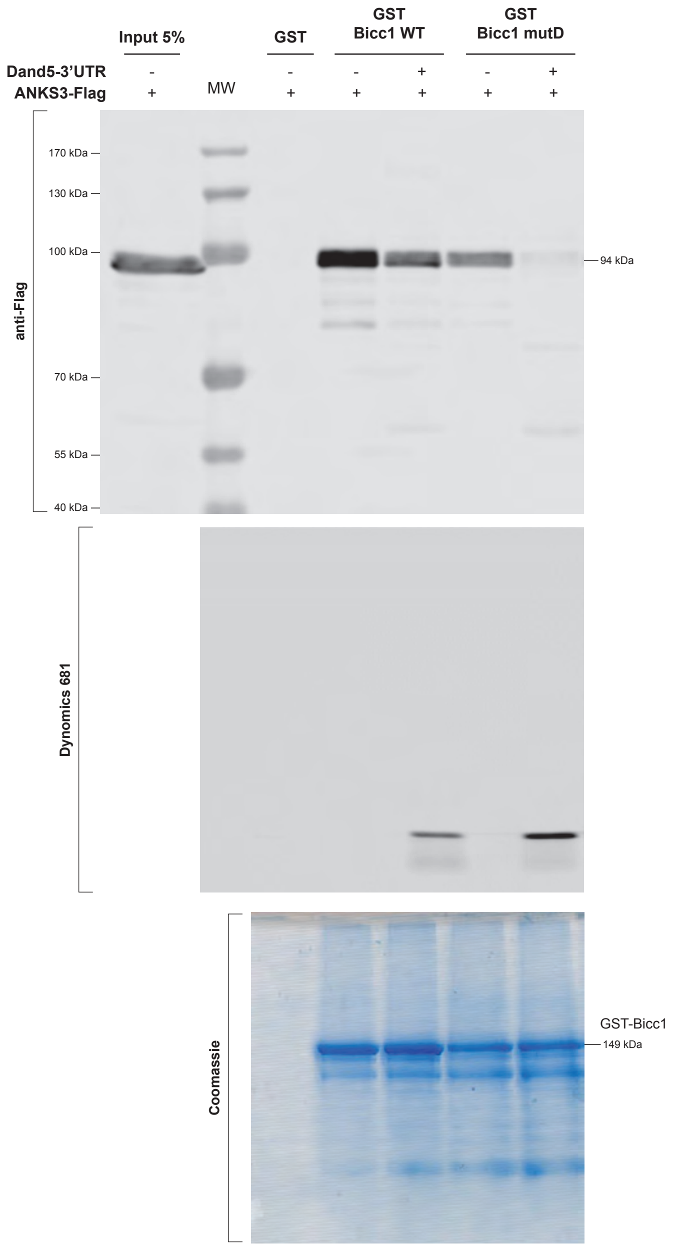

Figure 5 - source data

Figure 5B

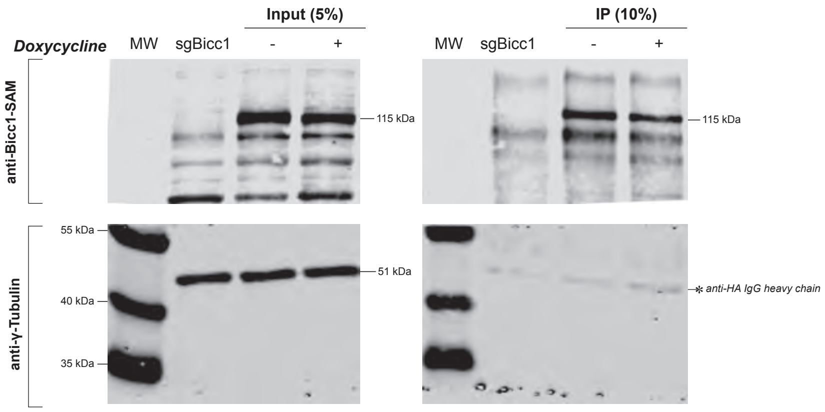

sgBicc1: absent on the main figure

Figure 6 - source data

Figure 6A

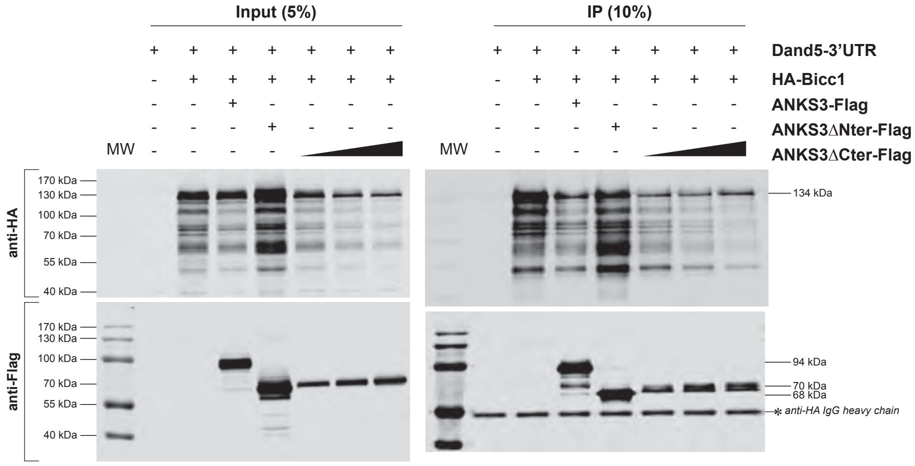

Figure 6B

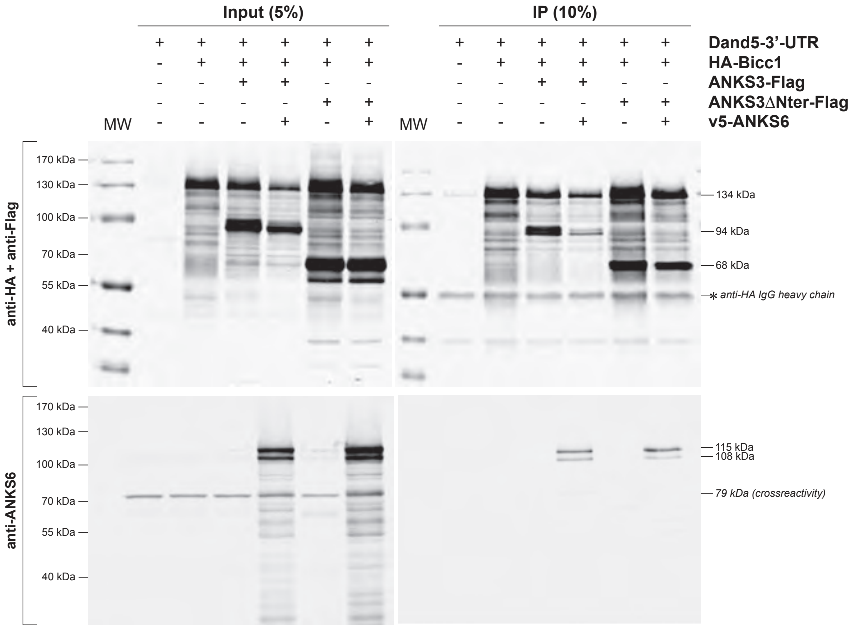

Figure S3 - source data

Figure S3A

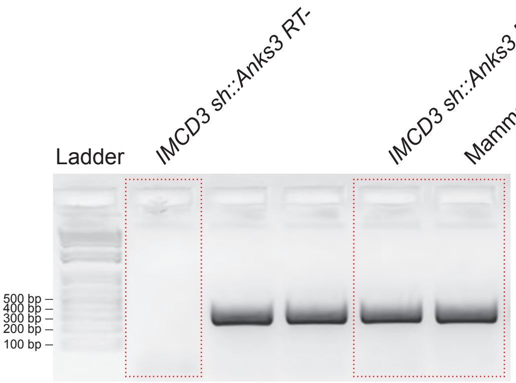

GAPDH
Expected size: 329 bp

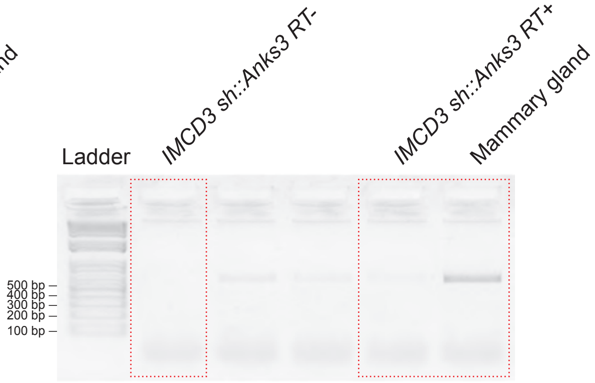

Dand5
Expected size: 580 bp

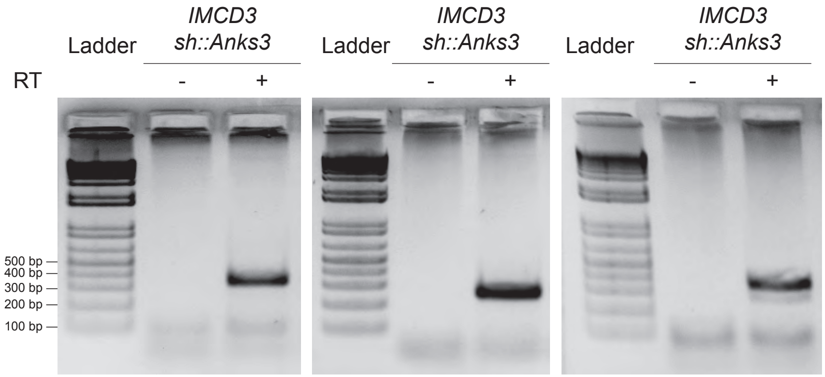

β-actin
Expected size: 364 bp

Bicc1
Expected size: 255 bp

Adcy6
Expected size: 324 bp
