# Supplementary material for: Bicc1 ribonucleoprotein complexes specifying organ laterality are licensed by ANKS6-induced structural remodeling of associated ANKS3
Source: PLoS Biol. 2023 Sep 21;21(9):e3002302. doi: 10.1371/journal.pbio.3002302 (PMC10513324; doi:10.1371/journal.pbio.3002302)
Supplement: Attachment — Submitted filename: renamed_a99e3.pdf [file pbio.3002302.s009.pdf]

Reviewer #1:

This manuscript by Rothé et al provides novel insights into the regulation of the RNA binding protein Bicc1, which has been implicated in mediating ciliopathy-associated phenotypes in animal models. The study focuses on the previously identified Bicc1 interacting proteins Anks3 and Anks6. Anks3 knockout mice show reduced expression of the Bicc1 target Dand5, and subsequently develop laterality defects. These results suggest Anks3 antagonizes Bicc1 activity. The authors then use modeling and multiple biochemical approaches to interrogate the interactions between Bicc1, Anks3, Anks6, and mRNA. Convincing results from these experiments support the authors' model that Anks3 inhibits Bicc1 from binding target mRNAs and that recruitment of Anks6 alters Anks3 conformation to promote Bicc1 interaction with mRNA. The authors propose an intriguing testable model in which availability of Anks6 that modulates Bicc1 is regulated via localization/sequestration in primary cilia. Overall, the manuscript is well written and the data are of high quality and clearly presented. The use of statistical analysis is appropriate. The findings are significant in that they shed light on molecular mechanisms of mRNA regulation in contexts that are relevant to human diseases. I have only minor comments for the authors to consider:

1) In the first paragraph of the Introduction section, I suggest citing recent work from the Hamada lab (Katoh et al, Immotile cilia mechanically sense the direction of fluid flow for left-right determination. Science. 2023 Jan 6;379(6627):66-71) to provide additional support for the statement "…stimulation of primary cilia by a leftward fluid flow accelerates the decay of Dand5 mRNA..."

We thank the reviewer for this suggestion. We now added the reference Katoh et al., 2023 on page 3 (first paragraph).

2) In the second paragraph of the Introduction section, I suggest spelling out sterile α motif (SAM), which currently appears in the third paragraph.

Since SAM is already spelled out in the abstract, we now removed this repetitive information from the third paragraph of the introduction.

3) From the analysis of laterality defects in Anks3 knockout mice, it is implied that only homozygous mutants present laterality defects since heterozygous animals were included in 'controls.' If indeed laterality defects were not observed in heterozygous mice, this should be clearly stated. On the other hand, a low incidence of laterality defects would also be interesting.

We now clarified on page 5 that no defects were observed in heterozygotes: "Heterozygous $Anks3^{+/\Delta ex10\text{-}11}$ mice derived from targeted cells appeared phenotypically normal.".

4) In the Results section titled 'Endogenous ANKS3 buffers the binding of Bicc1 to specific target mRNAs in IMCD3 cells,' I suggest changing 'in vivo' to 'in cells' in the sentence "To test whether ANKS3 and RNA compete for Bicc1 binding in vivo, we analyzed endogenous Bicc1 RNPs…"

The revised text now states: "To test whether **endogenous** ANKS3 and RNA compete for Bicc1 binding in **cells**".

Reviewer #2:

The manuscript by Rothé et al., presents embryological and biochemical data on the regulation of the posttranscriptional properties of Bicc1 by Anks3 and Anks6 with respect to Dand5 mRNA. Like in other vertebrate species, Anks3 loss-of-function in mice resulted into impaired organ laterality, which was caused by failed LR patterning. In Anks3 mutants, Nodal expression in the left lateral plate mesoderm was either lost or ectopically induced on the right. Anks3 function in laterality determination was further pinpointed to the process of symmetry breakage at the node. Judged by a transgene, Bicc1 dependent Dand5 asymmetry, which is the consequence of cilia-driven leftward flow, was no longer observed. The authors interpreted this result as precocious symmetric decay of Dand5 mRNA which was triggered by Bicc1. Therefore, Anks3 could serve as a Bicc1 regulator during symmetry breakage. Using structure prediction by Alphafold and numerous complex biochemical experiments, the authors dissect the molecular basis of Bicc1 interactions with Anks3 and Dand5 3'UTR. Surprisingly, the RNA binding domain of Bicc1 specifically interacts with the C terminus of Anks3 and thereby interfered with Dand5 mRNA binding. Further, the authors show that inhibition of RNA binding to Bicc1 by Anks3 was prevented by the presence of Anks6. A Bicc1 / Anks3 / Anks6 interplay had been established by the Constam group in the context of Bicc1 polymerization into granules. The new data now shows the effect of Bicc1 with respect to one of its target mRNAs. As such the manuscript is highly relevant and would fit to the scope of PLOS Biology. However, I do have some major and minor issues, which need to be addressed.

Major issues:

- The fundamental problem of the manuscript lies in the disconnect of embryological and biochemical data, which impede a coherent story. The analysis of the Anks3 mouse mutant provides no experimental evidence which underscores or matches with the demonstrated mechanism of Bicc1 / Ansk3 / Anks6 interaction. Moreover, the presented mouse data can be considered as preliminary and some of the conclusions are at least questionable.

The mouse data indeed focus on the analysis of organ laterality defects of a first *Anks3* mutation in a rodent model, and of the dsVenus-Dand5 3'UTR reporter transgene as a validated direct target of Bicc1 *in vivo*. Our data show that *Anks3* is required to protect this 3'UTR against bilateral repression, and to normally specify organ laterality. As these results precisely match the prediction by the mechanism of ANKS3-mediated inhibition of Bicc1 observed *in vitro* data, we cannot see how an alleged lack of such a match could be "the fundamental problem" of the manuscript.

1. As the authors noted, it is not clear if the CRISPR introduced deletion results into a null allele. Because the retained N-terminal sequence of Anks3 contains the ANK domain, a truncated protein could be expressed and functional the phenotype of a "true" knockout could differ. The lack of a kidney phenotype is an indication of retained Anks3 functions in the CRISPR mutant. However, clarifying this issue would be beyond the scope of the manuscript. The authors could test the corresponding N-terminal Anks3 sequence in their experimental setups and thereby provide some evidence on the potential activity of the mutant protein. In any case, the authors should take this circumstance into account and should not label the homozygous mutants as knockouts.

We would like to thank the reviewer for pointing out that it is important to not call the *Anks3* mutation a knockout. We agree, and now refer to this allele as $Anks3^{\Delta ex10\text{-}11}$ instead. This being said, our finding that ANKS3 attenuates Bicc1 binding to target mRNAs does not necessarily imply that it must be essential also in kidneys. A recessive mutation in human *ANKS3* associated with laterality defects did not result in kidney malformations either (Shamseldin et al., 2016), and *Anks3* knockdown in zebrafish by morpholinos led to pronephros dilatations at only a relatively low penetrance of 7 to 30% (Yakulov et al., 2015). Whether such pronephric cysts form independently of Bicc1 or due to a change in Bicc1 activity could be interesting to investigate in future studies. However, it should be noted that Bicc1 is a protective factor that inhibits cyst formation in the kidney, and that lack of inhibition by ANKS3 thus may increase rather than diminish Bicc1-mediated protection against cystic growth. In line with this prediction, forced overexpression of Bicc1 mRNA has not been reported to impair development of the pronephros in *Xenopus* (Tran et

al., 2010). Nevertheless, thanks to this helpful comment of the reviewer, we decided to remove our statement about the apparent absence of kidney cysts in newborn *Anks3* mutant mice to avoid a possible misunderstanding that we rule out any potential kidney phenotype. Indeed, based on our superficial analysis of organs documented in the supplemental Table S1, we cannot and do not rule out that some cysts might form only rarely without enlarging the kidneys, or only at later stages that we could not analyze due to the perinatal lethality.

2. The authors suggest that the Anks3 mutants somewhat phenocopies Dand5 knockout embryos. However, Dand5 knockout embryos show higher frequency of bilateral Nodal expression and prolonged Nodal expression was not reported. Later observation in Anks3 mutants may hint to reduced Lefty expression and/or disrupted midline.

dsVenus fluorescence remained significantly expressed even in *Anks3*$^{\Delta/\Delta}$ mutants, indicating that inhibition by the *Dand5* 3'UTR is considerable, but incomplete. Accordingly, we do not claim that Bicc1 should block Dand5 100%, as required to phenocopy *Dand5* null mutants. Secondly, while the reviewer may have information that *Nodal* expression does not persist in *Dand5*$^{-/-}$ null embryos, a partial inhibition of *Dand5* expression actually does indeed associate with prolonged *Nodal* expression also in *inv/inv* mutants, similar to what we observed here in *Anks3* mutants (Oki et al., 2009). We believe that these observations support our model rather than questioning it. Finally, the prolonged *Nodal* expression in *inv/inv* mutants was actually mediated by an increase in Lefty, rather than by a decrease (Oki et al., 2009). But even if an unknown mechanism were involved other than increased Lefty expression, this would not falsify our model either. In light of these considerations, we did not consider it sufficiently informative to examine Lefty expression also in *Anks3* mutants.

In consequence, Anks3 likely has more functions during LR development.

Anks3 mutants were analyzed here specifically to test whether the *Dand5* 3'UTR reporter, a genetically validated Bicc1 target mRNA, is also regulated by ANKS3 *in vivo*. The results support our model. We agree with the reviewer that one can never rule out additional functions. To acknowledge this, we stated in the Discussion (3rd paragraph): "Mechanistically, our analysis …revealed *an* essential function of ANKS3 to antagonize the mRNA decay…". To emphasize more strongly that additional mechanisms could also be involved, we revised the concluding sentence to now state: "*While these findings do not rule out additional unknown functions,* they support a role of ANKS3 in inhibiting Bicc1 activity."

3. In Anks3 mutants, precocious symmetric decay of Dand5 mRNA was suggested to be the cause of LR defects. This reasoning was deduced from a transgene, which drives expression of a destabilized Venus protein in the crown cells of the node. The corresponding transcript of the transgene is under the control of the Dand5 3'UTR. Besides representing only an indirect evidence, the timepoint of the analysis was too late to draw that specific conclusion. At 3-5 somite stage embryos, leftward flow was already sensed, which is demonstrated by Dand5 asymmetry. Hence, bilateral reduction of Dand5 could be the cause of a turbulent flow, which triggers Dand5 inhibition / decay in left and right crown cells (see 4.). Precocious Dand5 decay should be detectable at stages where leftward flow was not established yet or just started. Based on experiments of the Hamada group, first Dand5 asymmetries are already detectable at early headfold (EHF) stage. Dand5 asymmetries are basically penetrant as soon as somites form. In addition, blocking flow with methylcellulose at different stages, revealed that flow at EHF, which is weak and slow, is sufficient to determine laterality (Shinonara et al., 2011). Therefore, analysis of endogenous Dand5 mRNA expression prior somite stages needs to be presented to proof precocious symmetric decay.

We are greatly indepted to the reviewer for making us aware that our use of the term "precocious" was prone to a grave misunderstanding, and that the intended meaning of our statements is only conveyed with sufficient clarity if we actually drop the word "precocious". It is "precocious" on the R side, and only in the sense that Dand5 normally is not downregulated there in wild-type embryos until much later (>7 somites) stages. In other words, rather than claiming that Bicc1 is activated too early, our intended meaning is that *Anks3* mutants downregulate the *Dand5* 3'UTR even on the

RIGHT side already at a time when normally such downregulation is only seen on the LEFT. Accordingly, in all three instances (Abstract, Results and Discussion), the revised text now simply refers to "bilaterally symmetric" downregulation instead of calling it "precociously bilaterally symmetric".

Regarding the exact time of onset of the earliest *Dand5* mRNA decay, and the robustness and penetrance of the ensuing asymmetry, we are aware of only one study (from the Belo lab, not Hamada group) showing an embryo with asymmetric *Dand5* already at headfold stage (E7.5) (Marques et al., 2004). By contrast, the Hamada group detected no such asymmetry even at late headfold stage (see Fig. 4E in Oki et al., 2009). Furthermore, they showed that an emerging asymmetry is still not fully penetrant even as late as at the 3-somite stage (Minegishi et al. 2021). In the same figure of that same study (Fig. 1b, depicted here to the right), the Hamada group also validated that this asymmetric *Dand5* expression and its dynamics are faithfully recapitulated by the *Dand5* 3'UTR reporter transgene. Hence, they stated: "Both *dsVenus* mRNA (Fig. 1c) and dsVenus fluorescence (Fig. 1a and d) were bilaterally equal at the early headfold and zero-somite stages but were asymmetric (R > L) at the three- and five-somite stages, recapitulating the pattern of Dand5 mRNA (Fig. 1b and e)" (Minegishi et al., 2021). In other words, flow sensing during headfold stage in all of these later studies did not translate into significantly asymmetric *Dand5* expression at the node until after early somite stages.

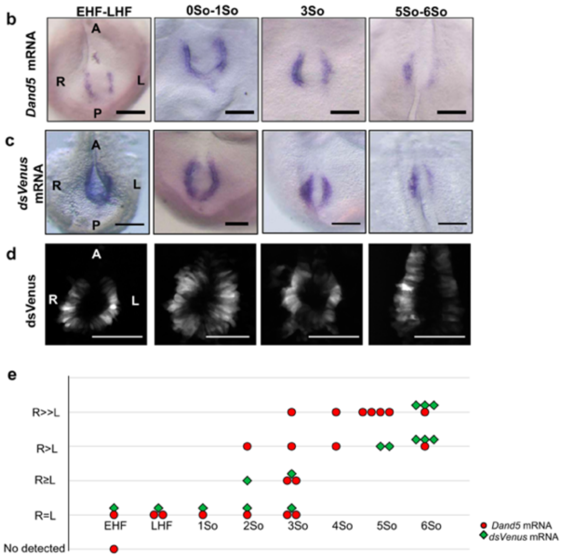

To us, this makes sense because we cannot think of a mechanism of how flow sensing at headfold stage would translate into robust asymmetric accumulation of any molecules such as *Dand5* mRNA without delay. Therefore, and since the *Dand5* asymmetry mediated by Bicc1 in wild-type embryos is not fully penetrant until the 3-somite stage, our analysis at 3-5 somite stage is indeed the correct stage to test if *Anks3* is important to protect the *Dand5* 3'UTR on the RIGHT side. Our results confirmed this prediction of our model.

The reviewer also pointed out that the evidence that *Anks3* regulates the asymmetric mRNA decay mediated by the *Dand5* 3'UTR is indirect, because we focused on the 3'UTR reporter rather than on endogenous *Dand5*. While this is correct, we believe that as a previously validated readout of Bicc1 activity, this reporter is actually superior to reduce the risk of indirect effects, because it contains only the short fragment with the validated Bicc1 target sequence. This considerably reduces the probability that the effect could be indirect, mediated e.g. by miRNAs, post-translational modifications, or proteins other than Bicc1 itself that could potentially act on *Dand5* mRNA only indirectly via regions that do not bind Bicc1. Imaging dsVenus is also superior to an analysis of endogenous *Dand5* mRNA in terms of the reliability of its quantification.

4. Anks3 and 6 have a record of being bona fide ciliopathy genes. Loss of Anks6 can alter cilia polarity. Planar polarity of ciliated LRO cells is a prerequisite to generate a directional flow. Impaired cilia polarity can create turbulent flow, which could lead to symmetric Dand5 downregulation. Therefore, cilia parameter like length and polarity needs to be assessed in Anks3 mutants. Flow analysis could provide data which solves this question, as well. In this context, Bicc1 could still be the critical target for Anks3, as Bicc1 loss-of-function impacts on cilia polarity, as well.

The nature of organ laterality defects and the *Nodal* expression pattern imply that the cilia of *Anks3* mutants still generate flow. The possibility that a more subtle cilia defect could result in "turbulent flow" was not investigated for several reasons, including:

i) Although potential effects of *Anks6* on cilia have been studied in multiple tissues across different model organisms, cilia polarity was found to be minimally perturbed, and only in multiciliated epidermal cells in *Xenopus: "Analysis of cilia in various tissues and model systems did not detect a role for ANKS6 in cilia formation or length control, but knockdown in Xenopus epidermal cells resulted in a mild defect in polarized orientation (Supplementary Fig. 9)." (Hoff et al., 2013)*. The same group also studied ANKS3 but reported no effects of ANKS3 on cilia or their polarity either. While we cannot rule out the possibility that these authors could have missed something, this seems unlikely, given their track record and expertise.

ii) In a separate study to be published elsewhere, we found that also the *Anks3* knockdown in IMCD3 cells did not impair the formation of cilia or their ability to enrich Anks6 in their inversin compartment. These results do not support a link of ANKS3 to inversin that might in turn impinge on cilia polarity.

iii) "Turbulent" flow was described in *inv/inv* mutants, where it inverts LR patterning rather than randomizing it as in our *Anks3* mutants (Okada et al., 1999). Likewise, computational modelling of the advection and vertical diffusion of a morphogen at the node suggests that slow flow (formerly called turbulent) would invert asymmetric nodal signaling rather than randomizing it (Cartwright et al., 2008). Turbulent flow in *inv/inv* mutants also did not abrogate the asymmetry of Dand5 as observed in *Anks3* mutants, but instead inverted it (Oki et al., 2009). In our opinion, these observations do not support the hypothesis here and in point 3 of the reviewer that nodal flow in *Anks3* mutants could be turbulent. Furthermore, even in the unlikely scenario that nodal flow in our *Anks3* mutants is turbulent, this would not rule out our model that Bicc1 could nonetheless be a direct target of ANKS3. Therefore, we believe the suggestion to rule out an unlikely defect in cilia lengthening or polarity is beyond the scope of this study.

- Posttranscriptional autoregulation by Bicc1 is an interesting feature, although a connection to the overall story is not obvious. An established Bicc1 target mRNA would be more qualified to be experimentally compared to the Dand5 3'UTR. If I got it correctly, HA-Bicc1 binds to its own mRNA. However, because the HA-Bicc1 expression vector does not contain the Bicc1 3'UTR, HA-Bicc1 binding must occur via the coding sequence of its mRNA. To my knowledge, Bicc1 generally acts via the 3'UTRs of target mRNAs. Thus, this observation is intriguing and I was wondering if something related has been reported before.

HA-Bicc1 binds its own mRNA, as shown by Piazzon et al. 2012, and here in figure 6A. Moreover, as another established Bicc1 target mRNA, we did actually analyze also endogenous Bicc1 binding to *Adcy6* mRNA in IMCD3 cells. The results show that it increases upon ANKS3 knockdown as predicted by our model (**Figs. 5 and S3**). However, we realize that our statement on p9 that "known Bicc1 targets include its own transcripts" was apparently easy to overlook. We therefore replaced it by a separate sentence to describe the rationale for analyzing the binding of endogenous Bicc1 to its own mRNA in more detail as follows: "…Therefore, we instead analyzed if *Anks3* knockdown alters the binding of endogenous Bicc1 to *its own transcripts or to endogenous Adcy6 mRNA, two alternative known targets in mammalian cells [9], and/or their expression.*"

A deletion of the KH domains that abrogates binding to RNA does not increase HA-Bicc1 expression, suggesting that in this overexpression setting, there is no Bicc1 autoinhibition (Rothé et al., 2023). Furthermore, we here showed that neither our *Anks3*$^{\Delta/\Delta}$ mutation in mouse embryos nor *Anks3* knockdown in IMCD3 cells alter Bicc1 protein expression levels (**Figs. S1D and 5B**). Therefore, we believe that Bicc1 autoregulation is not within the scope of this article. However, *Anks3* knockdown in IMCD3 cells here led to an unexpected increase in *Bicc1* mRNA expression levels. To increase clarity, the revised **Fig. S3B** shows this result by comparing conditions with and without doxycycline, instead of a comparison to β-actin control mRNA that may have obfuscated the meaning of this data. Moreover, as there was no corresponding increase in Bicc1 protein, and since it was not yet possible to resolve this paradox, we now mention in the Discussion (3rd paragraph) that Bicc1 is known to inhibit its own expression in *Drosophila* egg chambers through the binding of its own 5'UTR (Chicoine et al., 2007), but that this sequence is not conserved in vertebrates. One possible scenario is that binding of Bicc1 to other regions stabilizes its mRNA

in complexes that are not translated. Binding of mouse Bicc1 to its own mRNA neither requires the 5' nor the 3'UTR (Piazzon et al., 2012). Binding to *Dand5* and *Adcy6* mRNAs is mediated by their proximal 3'UTRs. Therefore, and since validated target mRNAs with known Bicc1 binding regions are scarce, one cannot conclude that Bicc1 generally binds via 3'UTRs.

In addition, this result raises the question to which extent such Bicc1 binding to its own coding sequence is functionally relevant and whether there is a general interference in Bicc1 gain-of-function experiments. I envision that HA-Bicc1 mRNA serves as a constant competitor to other mRNA targets i.e. Dand5 3'UTR. Does this issue has an impact on the obtained results? Which sequence in HA-Bicc1 mRNA gets bound by HA-Bicc1?

Most RNA-binding proteins interact with multiple targets, suggesting that competition among them would indeed not be unexpected. Therefore, and since competition among RNA for a shared binding protein is rarely studied, we agree that our data indicate that this interesting question could be a fruitful area for future investigation. However, it would not alter any of our conclusions since our gain-of-function experiments using HA-Bicc1 focused on HA-*Bicc1* and *Dand5*-3'UTR mRNAs that were both overexpressed to mitigate the risk of potential bias from competition.

Furthermore, competition among different RNAs for overlapping binding sites on one or several KH domains of endogenous Bicc1 would not introduce a technical bias either because in this setting, it would not be an artifact. To the contrary, binding of Bicc1 to its own transcripts would be well suited as another physiological buffer besides ANKS3 to increase the threshold of binding to specific target RNAs as well as to reduce access of non-specific mRNAs. However, testing this attractive hypothesis is beyond the scope of the present study and presently not feasible for technical reasons. In particular, mapping the Bicc1 binding region(s) in the coding region of *Bicc1* mRNA without disrupting Bicc1 function will not be straightforward because candidate regions will have to be provided in *trans* to validate the binding specificity by mutagenesis. Therefore, we chose to only discuss the possibility of competition (third paragraph of the Discussion). To address the question of the review, we expanded this paragraph and tried to increase its clarity.

As mentioned by the reviewer, Bicc1 binds to the coding sequence of its own mRNA. Despite many efforts, we could not identify a precise sequence, suggesting that the binding site is likely a composite of several short motifs distant from one another. This is indeed permitted by the mode of RNA recognition by arrays of KH domains, where each KH domain requires only 4 single-stranded nucleotides.

- Recently, the authors have shown an Bicc1 / Anks3 / Anks6 interaction in the context of Bicc1 granulaes. How does granulae formation contribute to the observations on Dand5 3'UTR binding by Bicc1?

To not unnecessarily complicate the story, we decided to report on the relationship with phase separation in a separate study (Rothé et al., 2023). In short, the ΔSAM mutant which cannot assemble granules still binds this and other target mRNAs such as *Adcy6* and *Bicc1* mRNAs (Piazzon et al., 2012). Conversely, Bicc1 ΔKH still forms condensates even though it does not bind RNA. Together, these observations imply that *Dand5* mRNA binding is neither the cause nor the consequence of Bicc1 condensate formation.

The mutant Bicc1bpk mouse line does not display LR patterning defects and this mutant Bicc1 protein is unable to polymerize. Is this mutant protein behaving identical to wt Bicc1 when used in the biochemical assays? Such data set would establish a stronger link to the formation of the LR axis.

There are several mutually non-exclusive possible explanations why *bpk* mice may lack LR patterning defects: Firstly, the *bpk* mutation affects only one of two alternatively spliced *Bicc1* transcripts (Cogswell et al., 2003). The second isoform remains competent to self-polymerize in cytoplasmic granules and to silence mRNA (Rothé et al., 2015), suggesting it may specify LR

asymmetry even if it is not sufficiently expressed or active at later stages to compensate for the major isoform in suppressing kidney cyst formation.

Furthermore, while HA-Bicc1^{bpk} fails to self-polymerize, its concentration in cytoplasmic granules was rescued when ANKS6 was co-expressed with ANKS3 (Rothé et al., 2018). Importantly, the bpk mutation also did not prevent binding of full-length Bicc1 to ANKS3 or to complexes of ANKS3 with ANKS6 (Rothé et al., 2018). Therefore, we expected that it should also not abrogate ANKS/ANKS6-mediated regulation of Bicc1 binding to target mRNAs. At the request of the reviewer, we now tested this prediction by expressing HA-Bicc1^{bpk} and the dsVenus-Dand5-3'UTR reporter alone or with ANKS3-Flag ± V5-ANKS6 in HEK293T cells. As shown below, ANKS3 inhibited the binding of HA-Bicc1^{bpk} to both its own mRNA and to the *Dand5*-3'UTR reporter, and ANKS6 still efficiently antagonized ANKS3 despite the *bpk* mutation. This confirms that the bpk mutation inhibits the extension of the Bicc1 SAM polymer, but without abrogating the SAM:SAM dimerization with ANKS3 that helps to antagonize RNA binding also in cell-free pull-down assays (**Fig. 4C-D**).

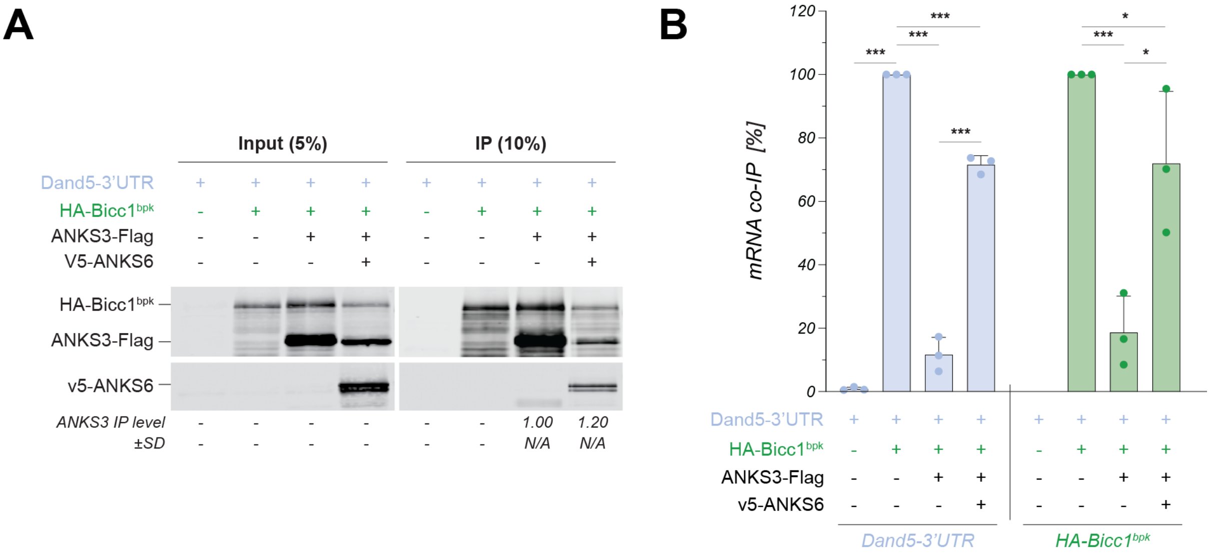

ANKS3 and ANKS6 also regulate the RNA binding activity of mutant HA-Bicc1^{bpk}. (A) *Western blots of HA-Bicc1^{bpk} immunoprecipitates and their inputs from extracts of HEK293T cells expressing the dsVenus-Dand5-3'UTR reporter and HA-Bicc1^{bpk} alone or in combination with ANKS3-Flag and v5-ANKS6.* ***(B)*** *Percentage of co-immunoprecipitated Dand5-3'UTR and HA-Bicc1 transcripts normalized to their amounts in inputs and to HA-Bicc1^{bpk} protein in the IP, relative to the corresponding condition with HA-Bicc1^{bpk} only. Data are means + SD from three independent experiments. N/A: not applicable; *p < 0.05, **p < 0.01, ***p < 0.001 (Student's t-test).*

While these results strongly corroborate our conclusions, we chose not to include them in the revised manuscript because we feel that they would unnecessarily complicate the story, given that LR patterning in *bpk* mutant mice to our knowledge has not been analyzed or described in the literature.

Minor points

- Italic letters for genes and transcripts needs to be applied throughout the text. Currently, italic style for mRNAs / genes is rather randomly used.

Indeed, the consistent use of italics is important in this study where proteins and their transcripts need to be clearly distinguished from one another. We tried to remove all remaining inconsistencies in our use of italics.

- Figure 3B: The nature of asterisk is not easily recognizable. The artefact could be directly marked.

We now placed the asterisks directly on the "shady band", together with a note in the legend stating: *"Note that GST-KH was so abundant in bead eluates that it is faintly visible also in the anti-Flag Western blot as a shady band closely above ANKS3 ΔNter marked by a blue asterisk"*.

- The different normalizations and calculations are difficult to follow. More detailed explanations in figure legends or in the method section would be surely helpful.

We thank the reviewer for pointing this out! In the submitted manuscript, we compared the effect of *Anks3* knockdown on Bicc1 RNA binding activity both before and after normalization to coIPs of β-actin mRNA as a non-specific control. We realized that our attempt to augment clarity by such transparency instead diminished it. To address this, the revised Fig. 5C now shows the level of non-specific binding to this control mRNA, and only without using this signal for an additional normalization step. However, the data remains fully transparent since the revised Fig. S3B still shows the impact of *Anks3* RNAi on the mRNA expression levels of the Bicc1 target mRNAs.

Besides simplifying the normalization, and to further improve this point, the revised Materials and Methods section now includes a separate paragraph entitled ***Quantification and statistical analysis*** to explain the normalization steps in each experiment (the text of that new section is included in our answer to point D of reviewer 3). We are very grateful to the reviewer for this suggestion because we believe it really helped to improve the clarity of this important result.

- The single cell data of early mouse embryos (Figure 1D) should be viewed as circumstantial evidence and removed from the main figure. The existence of Foxj1+ / Bicc1- / Dand5 + cells is particularly odd, as the authors show uniform Bicc1 expression in the node (Figure S1B). Bicc1 violine is shifted upwards.

For the reasons stated by the reviewer, we moved these data to supplementary Fig. S1E, and we now included also individual UMAP plots for *Dand5, Anks3* and *Anks6*.

Neither *Foxj1* (Brody et al., 2000), nor *Bicc1* (Wessely et al., 2001) or *Dand5* are expressed in any cells other than the node during the stages represented in the mesendoderm scRNA-seq dataset that was re-analyzed here. It makes sense that *Bicc1* reads were only detected in a subset of Foxj1+ cells, because node cells are marked by Foxj1 already before the onset of the earliest detectable *Bicc1* expression. This difference is likely amplified by the fact that node cells of early-streak stage mesendoderm were overrepresented (67%) in this scRNA-seq dataset compared to nodes from late-streak stages (Scheibner et al., 2021). For clarification, the revised text on p6 introducing these markers now states: *" Foxj1 and Bicc1 are specifically expressed in node cells, starting at mid- or late-primitive streak stages, respectively* (Brody et al., 2000; Wessely et al., 2001)*."* Our main conclusion that *Anks3* is indeed transcribed in *Bicc1*-expressing node cells is also further supported by the UMAP plot of *Anks3* that was initially missing.

We initially used Foxj1^{+}/Bicc1^{-} cells only to estimate if, in addition to the observed co-expression, of *Bicc1* with *Anks3* or *Anks6*, there might even be a correlation in their expression levels. While this appeared to be the case at least for *Anks6*, we now removed this comparison with apparently Bicc1-negative cells because it is not essential for our main conclusion. Furthermore, since the sensitivity of scRNA-seq to detect any given gene depends on sequencing depth, one should not infer from the absence of a signal in some cells that its expression was truly absent. However, the relevant conclusion here that both *Anks3* and *Anks6* mRNAs are detected in *Foxj1+/Bicc1+* cells remains unchanged.

Finally, the violin plot of *Bicc1* mRNA wrongly appeared to be shifted upward because the bottom of the violin below the cutoff of 1.0 UMI had been cropped away to highlight exclusively the cells that were defined as Bicc1+. To avoid the wrong impression that the violin might have been shifted,

the revised plot now shows the whole violin, and the selected cut-off of +1 is indicated instead by a stippled box.

- Is Anks3 expression detectable in node cells by in situ hybridization?

Our approach to re-analyze public single cell RNA-seq was motivated by the suggestion of a reviewer of an earlier version of this manuscript at another journal, and by the robustness of an available dataset tracing mesendoderm lineages at single-cell resolution in a time-resolved manner (Scheibner et al., 2021). Therefore, and in compliance with the 3R (Replace, Reduce, Refine) initiative, we decided to first use this public dataset, and only opt for yet another *in vivo*-based method if needed. Mouse *Anks3* cDNA and specific probes to detect *Anks3* or *Anks6* mRNAs by ISH have not been described, and obtaining the necessary animal permit to then first validate the specificity of probes detecting the appropriate splice variants would have been unrealistic within the context of a revision. Therefore, and since the *Anks3* and *Anks6* transcripts were detected in the population of interest in the published scRNA-seq dataset, we decided to not implement a different approach that is more prone to non-specific background. The UMAP plot of *Anks3* provided in the revised Fig. S1E confirms broad expression throughout mesendoderm, including Foxj1+ Bicc1+ cells.

- Figure S3: I am not aware of a report showing Dand5 expression in mammary glands. Please add reference when I am wrong.

As a positive control for *Dand5* expression, we initially considered mouse mammary gland because *Dand5* expression in murine 4T1 breast cancer cells promotes metastatic colonization (Gao et al., 2012). After confirming that *DAND5* is also expressed in a majority of human breast cancer cell lines and in myoepithelial cells of normal human adult mammary glands (https://www.proteinatlas.org/ENSG00000179284-DAND5/tissue), we validated that murine *Dand5* mRNA was also detected in mouse mammary gland cDNA that we received from the neighboring lab of Dr. Brisken at EPFL. Prompted by the request, we now state in the legend of Fig. S3 that mammary gland was chosen as a positive control "…based on a search of *DAND5*-expressing cell lines and tissues in the Human Protein Atlas database".

Reviewer #3:

The manuscript by Rothe et al. provides new mechanistic and biological insights into a crosstalk between Anks3, Anks6 and Bicc1. In particular the mouse data and the competition experiments are very compelling using overexpressed proteins are very convincing. Unfortunately, the loss-of-function studies and thus the in vivo significance of this crosstalk are not as well developed. Thus, whether finetuning of the levels of Anks3 and Anks6 is biologically critical is still lacing. In addition, there are several other aspects of the manuscript that require improvement.

Major Points:

A. Single Cell Studies:

1. The single cell analysis in Figure 1D must include feature plots for Anks6, Anks3 and Dand5. Only by including the feature plots can the reader really judge whether they are co-expressed. In fact, it is surprising that the authors have not provided a more traditional in situ-based support for the co-expression of these 4 RNAs/proteins using e.g. RNAscope.

We thank the reviewer for the important suggestion to include the UMAP plots for *Dand5*, *Anks3* and *Anks6*, which are now provided in the revised **Fig. S1E**. In addition, following the suggestion of reviewer #2, we also moved the violin plots to this supplemental figure.

We preferred scRNA-seq analysis because it is less prone to limited signal-noise ratios than hybridization-based techniques. As explained in our answer to the final minor points of reviewer #2, implementing and validating the specificity of these alternative *in vivo* approaches also would have been unrealistic for logistical reasons because the collaborating lab of Dr. Hamada who provided the *in vivo* analysis of *Anks3* in embryos has unfortunately closed down due to his retirement. By contrast, given the extremely localized onset of expression of *Bicc1* at the node (Wessely et al., 2001) and its combination with *Foxj1* as another highly specific marker of cilia-forming node cells (Brody et al., 2000), we consider the scRNA-seq data to be more conclusive, especially for a gene like *Anks3* which appears to be broadly expressed at least in the mesendoderm (**Fig. S1E**), since for broadly expressed genes additional controls would be needed to validate whether the ISH signal of any given probe is really specific.

2. The violin plots do not support the statement of the authors that Anks6 and Dand5 are higher in the Bicc1- cells. They appear very similar. Statistics need to be performed to support such a statement.

The Foxj1^{+}/Bicc1^{-} population was initially used as an additional control to try to assess whether *Anks3* or *Anks6* expression *levels* may correlate with those of *Bicc1*. However, since the sensitivity of scRNA-seq to detect any given gene depends on sequencing depth, we realized that we should not infer from the absence of a Bicc1 signal in a given cell that *Bicc1* expression was truly absent. Therefore, and since this comparison was not needed for the main conclusion, we removed the corresponding plot from the revised article, as well as the statement that *Anks3, Anks6* and *Dand5* are transcribed in the Foxj1^{+}/Bicc1^{+} cells "at elevated levels compared to Foxj1^{+}/Bicc1^{-} cells". Nevertheless, the only relevant conclusion that both *Anks3* and *Anks6* mRNAs were detected in Foxj1+/Bicc1+ cells remains unchanged.

3. The subclustering needs to be interpreted with caution. The annotation of the single cell cluster under investigation is "axial mesendoderm" and not all of the cells in this cluster are Foxj1+/Bicc1+ crown cells. The Foxj1+/Bicc1- cells may be of a completely different types and thus the authors would be comparing two unrelated cell types. Thus, this comparison needs to be removed from the manuscript, or supported by a more in-depth validation by e.g. RNAscope.

As suggested and as mentioned above, we removed this comparison from the revised manuscript. In the revised Fig. S1E, we also tried to improve the clarity by indicating more clearly that the violins represent cells in the Foxj1+ Bicc1+ population.

4. Finally, the methods section on the single cell data analysis needs to be expanded so that the reader can adequately judge how the authors reached their conclusions. Just referring to the Github page is insufficient.

We thank the reviewer for pointing out that the description of our reanalysis of the Scheibner et al. dataset was incomplete. In the revised version of the manuscript, the Materials & Methods section has been updated with further information.

B. RIP Loss-of-Function Studies:

1. In Figure 5C and D are confusing. They are the same data but normalized in different ways. This raises the question about the authors' interpretation. Providing two options, suggests that the "corrections" influence the data. Thus, the authors need to show the raw data of this experiment, i.e. the non-normalized and not corrected levels in the presence (one column) or absence of Dox (second column) for each of the three genes of interest (Bicc1, Adcyd6 and Ddx5) and the b-Actin control in the main figure. It is important that the readers can judge the unmanipulated results. Finally, while there is value in the "corrected" data, the authors need to decide, which one of the manipulations yields the most accurate representation of the data and this should be part of the main figure (in addition to the raw data). The other panel should be relegated to the supplement. Finally, it is unclear why the authors normalize to the amount of IP'ed Bicc1 protein. In the description of the previous panel, they stated that "[…] without changing Bicc1 protein levels (Fig. 5B)." Thus, Bicc1 levels should not be a factor and no correction for this is needed.

We initially used both the condition without Dox as well as the amount of coimmunoprecipitated *β-actin* mRNA as non-specific control for signal normalization (former Fig. 5C). Since we found that the level of the *Bicc1* mRNA significantly increased in *Anks3*-depleted inputs, providing an additional graph of the amount of bound transcript without normalization to its input level was important to ensure that the observed increase of *Bicc1* mRNA in the Bicc1 protein IP is not obscured by this signal normalization artifact (former Fig. 5D). However, since *Anks3* knockdown clearly enhanced the total amounts of both *Bicc1* and *Adcy6* transcripts in the Bicc1 IP but not *β-actin*, we agree that the different normalization in Figure 5C and D and their descriptions were potentially confusing. Therefore, to clarify how the data was normalized, we now followed the recommendation to simplify the revised **figure 5C** and focus on only the data without normalizing them to both the input level and to *β-actin*. To further increase clarity, we now also show that fold changes induced by Dox are normalized to the baseline without Dox. This remaining normalization is standard practice and inevitable in this type of experiments. Each step of signal normalization is now also described in depth in a new method section entitled "*Quantification and statistical analysis*" (see below). As requested by the reviewer, the raw data and the detailed calculation are also available in the S2_raw_values file.

Our statement "…without changing Bicc1 protein levels (**Fig. 5B**)." referred to the observation that the increased *Bicc1* mRNA expression level upon *Anks3* depletion was not accompanied by a change in Bicc1 protein levels. In the revised version of the figure, we included a quantification of the amount of Bicc1 in the input and IP fractions relative to the untreated condition. The quantification confirmed that the depletion of *Anks3* does not enhance the level of Bicc1, which appeared even slightly reduced (fold change: 0.8x). In the IP fraction, the average amount of Bicc1 remained unchanged between the two conditions. However, as illustrated by a standard deviation of ±0.65, inevitable variation among individual experiments justifies the integration of the protein signal in our normalization.

We are extremely grateful to this reviewer for the suggestion to check our signal normalization for yet another reason: While revising the explanation of how the data were normalized, we noticed that the *Ddx5* mRNA coIP signals were accidentally normalized to the wrong column in our Excel

calculation sheet containing the values of IP/input ratios instead of raw IP values of *β-actin* control mRNA. After correcting this error, an apparent increase in *Ddx5* mRNA binding no longer reached statistical significance, even after repeating the experiment a fourth time. Therefore, and since Bicc1 has only been observed to bind the *Ddx5* 3'UTR when overexpressed in *Xenopus* (Zhang et al., 2013), we decided to remove *Ddx5* data from Figs. 5C and S3C and from the corresponding revised text. Instead, our statements are now limited to *Adcy6* and *Bicc1* mRNAs where our fourth repeat experiment confirmed that the depletion of *Anks3* significantly increases their association with endogenous Bicc1.

2. In the same paragraph, the final conclusion appears contradictory in respect to Adcy6. The authors state "By contrast, the accumulation of Adcy6 mRNA was unaffected, consistent with previous observations that it also remains unchanged upon binding to Bicc1 (Piazzon et al., 2012)." Then in the next sentence the state "Taken together, these results show that ANKS3 attenuates Bicc1 binding to Adcy6 and Ddx5 transcripts […]." So, is Adcy6 increased or not.

We apologize for this lack of clarity. Our results show that shAnks3 increases the binding of *Adcy6* mRNA to endogenous Bicc1 in coimmunoprecipitates from IMCD3 cells, and that it does so without altering *Adcy6* mRNA expression levels (accumulation). To clarify, we modified the first sentence as follows: “By contrast, **the expression level** of *Adcy6* mRNA was unaffected, consistent with previous observations that it also remains unchanged upon binding to Bicc1 (Piazzon et al., 2012)."

C. Anks3 Antagonism studies.

1. In Figure 6 the authors investigate a "possible antagonism between the effects of Nter and Cter regions of ANKS3 on the RNA binding activity of Bicc1. While the different activities of the two parts of the proteins are very clear, the Anks6 dCter is expressed at lower levels than the dNter (Fig. 6B). To rule out that the effect is more due to expression levels, the authors should perform the experiments with different concentrations of the Anks6 (we assume the reviewer meant Anks3) mutation constructs.

We would like to thank the reviewer for pointing out that the steady-state level of ANKS3 ΔCter in the input fraction is indeed below that of ANKS3 ΔNter. Therefore, and as suggested by the reviewer, we repeated the co-immunoprecipitation after increasing the dosage of ANKS3 ΔCter expression vector up to 4-fold. Importantly, quadrupling the dosage sufficed to enrich ANKS3 ΔCter in the coIPs to a similar extent as ANKS3 ΔNter. Despite this robust association with HA-Bicc1, ANKS3 ΔCter still entirely failed to impair HA-Bicc1 binding to the *Dand5*-3’UTR. These data corroborate our AlphaFold2 structure model that ANKS3 inhibits the RNA binding at least in part through an interaction of its Cter domain with Bicc1 KH domains. By contrast, they do not support the notion that the truncation of ANKS3 Cter enhances RNA binding only indirectly by diminishing ANKS3 expression.

As described in the initial version of this manuscript, the Cter domain of ANKS3 also inhibits the binding of Bicc1 to its own mRNA. Interestingly, when we increased the dosage of ANKS3 ΔCter, the inhibitory effect of the Cter became even more evident: Not only did the increase of bound ANKS3 ΔCter fail to inhibit HA-Bicc1 binding to its own transcripts, it even stimulated it above the baseline seen in the absence of ANKS3. These data strongly support our earlier conclusions that RNA binding of Bicc1 is inhibited by the ANKS3 Cter domain, and that this inhibition antagonizes a Bicc1-activating function of the ANKS3 Nter domain. In the revised version of the article, we therefore included these new data in the revised **figure 6A**.

In addition, we observe that while multiplying the dosage of ANKS3 ΔCter increased the binding of HA-Bicc1 to its own transcripts, binding to the *Dand5* 3'UTR remained unchanged, indicating that the latter reached its maximum already in the absence of ANKS3 ΔCter. The difference between these two distinct targets points to specific differences in their binding affinities and/or in their mode of binding (e.g. relative contributions of individual KH domains). However, we did not try to further distinguish between these and other scenarios because such differential regulation of

these two targets was only observed in the relatively artificial context of an overexpressed ANKS3 truncation mutant.

D. Experimental Details.

The authors need to provide more experimental detail on the amounts of constructs/proteins used. As the entire study is about stoichiometry, this information is critical for the data interpretation. The authors need to thoroughly go through the entire manuscript and amend the experimental details either in the Methods section or the figure legends.

We completely agree that stoichiometry is important. The Methods in the section "RNA co-immunoprecipitation assay in IMCD3 and HEK293T cells" therefore stated initially that*: "HEK293T cells were transfected with 2 µg Dand5-3'UTR, HA-Bicc1 and ANKS3-Flag plasmids each per dish, and 8 µg v5-ANKS6. HEK293T cells from two 10 cm dishes…"*. The same stoichiometric ratio was also used in our pull-down experiment. To further clarify this information, the revised Method section "Reconstitution of multiprotein complexes and RNP by GST pull-down" now states that "*HEK293T cells cultured in 10 cm dishes were transfected with 2 µg of ANKS3-Flag and 8 µg of v5-ANKS6 and extracted as described above. Cleared extracts corresponding to one third of a 10 cm dish per binding assay were incubated for 2 hrs at 4°C with glutathione-Sepharose 4B beads coated with GST alone (control), or with GST-Bicc1 fusions or RNPs*". In addition, to describe the new experiment of ANKS3 ΔCter titration mentioned above, we similarly updated the Method section entitled RNA co-immunoprecipitation assay by stating: *"For the experiment of ANKS3 ΔCter-Flag titration, double and quadruple doses of 4 µg and 8 µg per dish were used, respectively"*.

Finally, and as mentioned above and in our replies to Reviewer #2 (Minor point 3), the revised Methods now include a new section entitled "Quantification and statistical analysis" to describe how signals were normalized in each experiment.

Minor Points:

E. The statement in the discussion "Cystic growth in kidneys is stimulated by cAMP (Wallace, 2011), which also accumulates in Bicc1 mutant kidneys, likely due to de- repression of adenylate cyclase 6 (Adcy6) mRNA translation (Piazzon et al., 2012)." is an oversimplification of PKD. While Ca2+ plays a role it, it by far not the only pathway dysregulated in ADPKD. Thus, the authors should change this in lieu of one acknowledging the complexity of signaling impacted in PKD.

To not give the wrong impression to non-specialized readers that the cAMP/PKA pathway is alone responsible for cystogenesis, we revised this sentence as follows: *"Functionally relevant stimuli of kidney cyst formation in PKD include cAMP (Wallace, 2011). Cyclic AMP also accumulates in Bicc1 mutant kidneys…"*

F. The authors state that "Anks3-/- neonates showed no kidney cysts (unpublished observation), […]" These data should be included in the manuscript in the supplemental material, as this is important for the fact that the crosstalk with Anks6 and Bicc1 during L/R specification and kidney disease are different.

As mentioned in our reply to point 1 of Reviewer #2, our finding that ANKS3 attenuates Bicc1 binding to target mRNAs does not necessarily imply that its loss must necessarily evoke a disease phenotype also in kidneys. In the kidney, Bicc1 is a protective factor that inhibits cyst formation. Thus, lack of inhibition by ANKS3 may increase rather than diminish Bicc1-mediated protection against cystic growth. In line with this prediction, a recessive mutation in human ANKS3 associated with laterality defects did not result in kidney malformations (Shamseldin et al., 2016). However, since our *in vivo* analysis focused on early embryonic development, it examined kidneys only superficially at perinatal stages (Table S1). Thus, a potential subtle or incompletely penetrant impact of this or other *Anks3* mutations on kidney development cannot be formally ruled out at this stage without rigorous histological analysis of kidneys from a large number of animals.

Unfortunately, since kidneys of the neonates analyzed in Table S1 by the Hamada lab showed no overt overgrowth, they were discarded. Therefore, our claim that no kidney cysts develop has been removed from the revised manuscript.

REFERENCES

Brody, S.L. et al. (2000). Ciliogenesis and left-right axis defects in forkhead factor HFH-4-null mice. American Journal of Respiratory Cell and Molecular Biology *23*, 45–51.

Cartwright, J.H.E. et al. (2008). Fluid dynamics of nodal flow and left-right patterning in development. Dev Dyn *237*, 3477–3490.

Chicoine, J. et al. (2007). Bicaudal-C recruits CCR4-NOT deadenylase to target mRNAs and regulates oogenesis, cytoskeletal organization, and its own expression. Developmental Cell *13*, 691–704.

Cogswell, C. et al. (2003). Positional cloning of jcpk/bpk locus of the mouse. Mammalian Genome *14*, 242–249.

Gao, H. et al. (2012). The BMP Inhibitor Coco Reactivates Breast Cancer Cells at Lung Metastatic Sites. Cell *150*, 764–779.

Hoff, S. et al. (2013). ANKS6 is a central component of a nephronophthisis module linking NEK8 to INVS and NPHP3. Nature Genetics *45*, 951–956.

Katoh, T.A. et al. (2023). Immotile cilia mechanically sense the direction of fluid flow for left-right determination. Science *379*, 66–71.

Marques, S. et al. (2004). The activity of the Nodal antagonist Cerl-2 in the mouse node is required for correct L/R body axis. Genes and Development *18*, 2342–2347.

Minegishi, K. et al. (2021). Fluid flow-induced left-right asymmetric decay of Dand5 mRNA in the mouse embryo requires a Bicc1-Ccr4 RNA degradation complex. Nature Communications *12*, 4071.

Okada, Y. et al. (1999). Abnormal Nodal Flow Precedes Situs Inversus in iv and inv mice. Molecular Cell *4*, 459–468.

Oki, S. et al. (2009). Reversal of left-right asymmetry induced by aberrant Nodal signaling in the node of mouse embryos. Development *136*, 3917–3925.

Piazzon, N. et al. (2012). Bicc1 links the regulation of cAMP signaling in polycystic kidneys to microRNA-induced gene silencing. Journal of Molecular Cell Biology *4*, 398–408.

Rothé, B. et al. (2015). Bicc1 polymerization regulates the localization and silencing of bound mRNA. Molecular and Cellular Biology *35*, 3339–3353.

Rothé, B. et al. (2018). Crystal structure of Bicc1 SAM polymer and mapping of interactions between the ciliopathy-associated proteins Bicc1, ANKS3, and ANKS6. Structure *26*, 209-224.e6.

Rothé, B. et al. (2023). Antagonistic interactions among structured domains in the multivalent Bicc1-ANKS3-ANKS6 protein network govern phase transitioning of target mRNAs. IScience *26*, 106855.

Scheibner, K. et al. (2021). Epithelial cell plasticity drives endoderm formation during gastrulation. Nature Cell Biology *23*, 692–703.

Shamseldin, H.E. et al. (2016). ANKS3 is mutated in a family with autosomal recessive laterality defect. Human Genetics *135*, 1233–1239.

Tran, U. et al. (2010). The RNA-binding protein bicaudal C regulates polycystin 2 in the kidney by antagonizing miR-17 activity. Development *137*, 1107–1116.

Wallace, D.P. (2011). Cyclic AMP-mediated cyst expansion. Biochimica et Biophysica Acta - Molecular Basis of Disease *1812*, 1291–1300.

Wessely, O. et al. (2001). Identification and expression of the mammalian homologue of Bicaudal-C. Mech Dev *101*, 267–270.

Yakulov, T.A. et al. (2015). Anks3 interacts with nephronophthisis proteins and is required for normal renal development. Kidney International *87*, 1191–1200.

Zhang, Y. et al. (2013). Bicaudal-C spatially controls translation of vertebrate maternal mRNAs. RNA *19*, 1575–1582.
