# Supplementary material for: Bicc1 ribonucleoprotein complexes specifying organ laterality are licensed by ANKS6-induced structural remodeling of associated ANKS3
Source: PLoS Biol. 2023 Sep 21;21(9):e3002302. doi: 10.1371/journal.pbio.3002302 (PMC10513324; doi:10.1371/journal.pbio.3002302)
Supplement: Attachment — Submitted filename: renamed_c70f3.pdf [file pbio.3002302.s010.pdf]

Swiss Institute for Experimental Cancer Research
Prof. Daniel B. Constam

Reviewer remarks:

Reviewer #1: The authors have addressed all of my comments on the manuscript and have improved clarity. I find the revised manuscript appropriate for the audience of PLoS Biology.

Reviewer #2: The authors addressed most of my questions and based on the convincing biochemistry I support the publication in PLOS Biology.

In my view, one issue remains. The sole analysis of the Dand5 3'UTR transgene in Anks3 mutants is not sufficient to conclude that the mechanistic cause of LR defects is based on precocious decay of dand5 mRNA. Loss of transgene asymmetry and reduction of signals, at embryonic stages were flow has been already sensed, could be the consequence of two possible modes of Anks3 function: 1. flow independent, precocious decay of dand5 mRNA, as the authors propose and / or 2. turbulent flow, which results into bidirectional fluid motion and thus flow sensing at the right and left side of the node.

The reasoning for the second scenario is based on:

Turbulent flow has been reported in mouse and frog. Song et al., 2010 demonstrated that Wnt/PCP mutants have lost cilia polarity which resulted in a turbulent flow at the node and to left isomerism in mutant mice. Another example of turbulent flow was detected in Inv/Inv mutants. As stated by the authors, the development of Situs inversus in Inv/Inv mutants seems not congruent with flow pattern. However, Oki et al., 2009 demonstrated that the genetic background contributed to the phenomena. In a hybrid background, Inv/Inv mutants depict mostly bilateral activation of the nodal cascade, which matches with flow pattern. Most importantly, Bicc1 mutants also show defective cilia polarity and turbulent flow.

Based on the published record, a hypothesis where Bicc1 interacting proteins like Anks3 or 6 are required for cilia polarity and thus for directionality of leftward flow is reasonable.
The current analysis of the Dand5 3'UTR transgene in Anks3 mutants is not able to discriminate between both scenarios because predicted results are identical, i.e. loss of asymmetry and reduction of expression on the right.

The strong biochemical data, provided by the authors, suggests that precocious Dand5 decay in Anks3 mutants is indeed very likely but still the authors should tune down their wording about the in vivo situation. The acknowledgement and description of an alternative scenario does not diminish the relevance of the work and just reflects the complexity of Bicc1 functions during LR development.

School of Life Sciences
EPFL SV UPCDA
Prof. Daniel Constam
Station N° 19
CH - 1015 Lausanne
Phone : +41 21 693 07 41
E-mail : daniel.constam@epfl.ch
Website : https://constam-lab.epfl.ch

We thank the reviewer for appreciating that our biochemical data supports the proposed mechanism of how ANKS3 might protect the *Dand5* 3'UTR against Bicc1-mediated decay. At the top of p13 of the revised Discussion, we already acknowledge that our findings do not rule out additional possible functions for *Anks3*: "*While these findings do not rule out additional unknown functions, they support a role of ANKS3 in inhibiting Bicc1 activity.*" We also explained already in our answer to the previous point 4 why the alternative hypothesis of turbulent flow in *Anks3* mutants to our knowledge is not supported by available evidence, and this is not contested here by the reviewer. Furthermore, in the absence of an experimentally validated alternative, the most plausible mechanism of how turbulent flow could alter *Dand5* mRNA expression is by altering the availability of ANKS6 for activating Bicc1. If correct, the hypothesis of turbulent flow would be consistent with our model, rather than competing with it as an alternative. In PCP mutants described by Song et al., 2010, *Dand5* expression was not analyzed. However, our model predicts that the dynamics of ANKS6 activation in these mutants will likely be impaired due to impaired flow. Likewise, in *inv/inv mutants*, failure to sequester ANKS6 in the ciliary Inversin compartment (Hoff et al., 2013; Nakajima et al., 2018) can be expected to diminish the threshold required for flow to activate Bicc1 bilaterally, even regardless of the flow pattern, as observed in the alternative genetic background of inv/inv embryos analyzed by Oki et al., 2009. By contrast, in *Bicc1*$^{\Delta ex1/\Delta ex1}$ mutants, we previously described that "*…the leftward flow at the node was disrupted in half of the embryos (n = 5/9), with the other half (n = 4/9) showing normal leftward flow (Fig. 4f). Nonetheless, an artificial leftward fluid flow failed to rescue the pattern of dsVenus fluorescence in Bicc1*$^{\Delta ex1/\Delta ex1}$ *embryos examined (n = 7/7), suggesting that the mutant embryos are unable to respond to the fluid flow (Fig. 4g and h).*" (Minegishi et al., 2021). In summary, even in the unlikely event that flow were perturbed in *Anks3* mutants as hypothesized by the reviewer, it would not provide a Bicc1-independent alternative mechanism to explain their L-R phenotype. Therefore, we are not convinced that the purely hypothetical scenario of potentially impaired flow in *Anks3* mutants merits a separate discussion.

Reviewer #3 (Oliver Wessely, signs review): For the revised manuscript the authors have been very responsive to the reviewers comment. As a result the manuscript has been vastly improved. I still have two comments on the single cell data, which need to be addressed.

In response to the comments on the single cell data, the authors changed the text to "We found that Anks3, Anks6 and Dand5 are indeed transcribed in the Foxj1+/Bicc1+ cells (Fig. S1E)." Yet, this wording suggests that these genes are specifically expressed in the Bicc1-positive cells, which obviously is not the case. Thus, the authors should rephrase this sentence to something like "While Anks3, Anks6 and Dand5 are broadly expressed in the developing embryo, they are also expressed in the Foxj1+/Bicc1+ cells (Fig. S1E). They also should remove "likely" from the next sentence, because the scRNA-seq data do not provide a strong reason to perform the subsequent experiment, but instead support the author's hypothesis that this is a possible interaction.

We would like to thank again Dr. Wessely for his various constructive criticisms that we think helped considerably to improve the revised manuscript. However, *Dand5* expression is restricted to the node, and the claim that *Anks3* and *Anks6* are "broadly expressed" would require analysis in

EPFL SV UPCDA
Prof. Daniel Constam
Station N° 19
CH - 1015 Lausanne

Phone : +41 21 693 83 41
E-mail : daniel.constam@epfl.ch
Website : https://constam-lab.epfl.ch

lineages other than mesendoderm. Therefore, and since we nowhere claim that *Anks3* or *Anks6* are transcribed exclusively in node cells, we prefer to leave the current simple description of this result unchanged. We fully agree that a misunderstanding to the contrary is obviously ruled out by the data. To also explicitly clarify that node cells are indeed not the only mesendoderm cells that transcribe *Anks3* and *Anks6*, we instead revised the corresponding text in the Discussion by adding the words "including in…" in this sentence at the bottom of p12: "*…mining of public scRNA-seq data confirmed that Anks3 and Anks6 transcripts are detected in mesendoderm, including in cells that coexpress Foxj1 and Bicc1 mRNAs, two independent markers of ciliated node cells [26,27].*" We are also grateful for the suggestion to improve the corresponding conclusion statement in Results, where the revised text now omits the word "likely" and instead states: "*We found that Anks3, Anks6 and Dand5 are indeed transcribed in the Foxj1$^+$/Bicc1$^+$ cells (**Fig. S1E**), as expected if ANKS3 and its ability to recruit ANKS6 regulate the function of Bicc1."*

Finally, the methods section, is still very general and should be modified to actually describe how the panels, which are part of Figure S1E were generated.

We revised this section once more, particularly to further clarify that we simply used the same published Python command lines as the authors of this public scRNA-seq dataset that are readily available on the indicated github link.
